# Production Planning Using a Shared Resource Register Organized According to the Assumptions of Blockchain Technology

**DOI:** 10.3390/s23042308

**Published:** 2023-02-19

**Authors:** Barbara Balon, Krzysztof Kalinowski, Iwona Paprocka

**Affiliations:** Department of Engineering Processes Automation and Integrated Manufacturing Systems, Faculty of Mechanical Engineering, Silesian University of Technology, Konarskiego 18A Str., 44-100 Gliwice, Poland

**Keywords:** shared resource registry, production planning, blockchain technology, distributed network, internet of things, Industry 4.0

## Abstract

This article presents the architecture of integration of blockchain technology (BCT) and the Internet of Things with the planning of production processes. The authors proposed a shared concept of a distributed machine database based on BCT. As part of the work, a network of connections for the exchange of production resources was created using nodes communicating in a decentralized system, which at the same time serves as an integration of the virtual and real environment. Particular attention was focused on developing an algorithm for the efficient division of production tasks between all interested network users. BCT is used to conclude smart contracts and transactions and ensure the security of exchanged production data within shared ledgers. The proposed concept is a solution enabling a modern approach to the interdisciplinary management of production resources while maintaining the highest cybersecurity standards.

## 1. Introduction

The smooth, error-free and reliable flow of information regarding production resources both within the company and in relations with other companies is considered as one of the most important factors affecting the proper course of production processes. Constant attention to shortening production processes, care for the environment and the search for savings as well as the desire to combine traditional methods of production planning with modern solutions in the field of management applications contributed to the search for new production solutions.

The progressive digitization of the production, logistics and supply chain sectors is promoted by Industry 4.0. The development of larger factories and the slow takeover of market control has become a serious problem for the small business sector, which must compete with low prices and the global reach of the leaders in order to stay in business. The implementation of distributed digital platforms may allow developing companies to stay in the market and thus achieve a global reach and cooperate on equal terms with the recognized giants of the manufacturing industry. Dynamic changes in the production environment and the growing customer demands encourage manufacturers to interoperate, especially in the area of data management in the Internet of Things (IoT) environment. 

In the global reading, privileged access to natural resources, financial capital, qualified staff, and even the most modern machinery parks are not sufficient features to gain a competitive advantage and become market tycoons. In the light of market analyses and subsequent industrial revolutions, it seems necessary to look for solutions that will enable profitable production. It is becoming more and more popular to manage the enterprise in cyberspace and adopt decentralization as a new benchmark for data storage, processing and analysis, mainly to have a faster and professional insight into production lines. Such activities make production processes more efficient and individual manufacturing operations susceptible to immediate modifications. For the above factors, the area of IoT is extremely important and gives the opportunity to function in the blockchain (BC) network. 

BCT operates within the IoT. IoT is a network of interrelated objects with unique addressing characteristics that can interact with each other and collaborate with other enterprises to achieve joint or individual solutions [1]. This concept effectively integrates the IoT environment (Internet, cloud data, computers, network devices, simulations, tracking of production operations) with the real production environment (inventory management, storage and transport of products, production planning). This is thanks to professional access to production data in the cyberspace of the steering wheel, and managers have the ability to perform analysis, planning and scheduling and then make sound decisions in real time. 

Since IoT-based applications are constantly struggling with the problems of security and confidentiality of the data placed in them, the aspects of security and resistance to manipulation of data records are perfectly handled by blockchain technology (BCT). This technology is undoubtedly the direction of development in the automation of business processes without delays and guaranteeing security in accessing and exchanging production data. The flexibility of production systems and the ability to adapt in a dynamically changing manufacturing area are the trends that currently determine the direction of development of the technology sector. 

Another important challenge for manufacturers is the skillful storage, distribution and processing of valuable production data, which will not succeed without solutions ensuring the security, smoothness and accuracy of the transmitted information. The main task of BC technology is to eliminate the threat of product counterfeiting or to ensure the integrity of information from IoT devices [2,3]. In addition, many researchers have recognized that BC is the missing element of IoT functionality [4]. By combining BC technology with the use of IoT and artificial intelligence, production processes become more flexible and agile [1,5]. 

Following this lead, the main purpose of the article is to apply an architecture for a data exchange network between manufacturing companies, taking into account a shared data register with a particular emphasis on resources.

### 1.1. Literature Review and Related Research Work

The first literary mention related to the outline of BCT appeared thanks to Satoshi Nakamoto, who described the creation of the digital currency Bitcoin [5]. Another mention is made by Vitalik Buterin on the occasion of the network called Ethereum [6]. This network introduced smart contracts as new functional properties (protocols designed for the conclusion and automatic verification of digital contracts in accordance with predetermined conditions), which contributed to its identification as the underlying BC technology [5]. The beginnings of the BC idea date back to 1991, when the concept of use signed chains of information in the form of a tamper-proof e-ledger and a consensus model as goals in reaching agreements was created. The following years saw the evolution of the technology and its use in maintaining smart digital contracts [7,8]. In the field of BC, the most modern scientific research is focused on the optimization of technology and its synchronization with Industry 4.0 technologies, in particular IoT to support digital data storage [9].

The breakthrough came when IBM and Maersk announced a partnership to implement BC technology in the supply chain and integrate BC with IoT. They proposed a BC-based management platform for accurately recording and tracking aircraft spare parts, leading to an increased inspection accuracy inventory, reduced maintenance errors and efficient decision-making [10,11,12]. Kandah et al. proposed BC technology in smart cities and connected vehicles as an integrated system for distributed, tamper-proof and reliable trust management [13]. The interest in BC is particularly visible in industries related to medical documentation processes [14], legal services [15], data traceability [16] or the automotive industry [17]. A systematic literature review and explanation of the potential contribution of BCT to the economy, environment and society, and studies of manufacturers and their supply chains were carried out by Khanfar [18]. Jabbar reviewed the applications of BC in intelligent transport [19]. 

BC uses cryptographic concepts (one-way hash functions), asymmetric cryptography or time stamping. BC version 1.0 assumes decentralization in the transfer of value between entities in a trustless environment, and this mainly applies to applications that support cryptocurrency transactions. BC 2.0 expands intelligent connections and applications responsible for cryptocurrency transactions, especially in the economic and financial area [20]. BC version 3.0 adopts the main features of BC in trustless decentralization, e.g., data transparency in a decentralized network and immutability for developed web applications (e.g., currencies and investments) in line with the assumptions of BCT [21]. BC is one of the best ways to securely track online activity, update and secure data, and create a data history [22]. A regular database could be used to track previous transaction exchanges between two parties, but it is BC assumptions that can eliminate delays and improve connections [23,24,25,26]. For example, in supply chain tracking, several private BC networks connect their numerous partners and customers via a secured BC for data sharing and secure payments [27]. In a decentralized system, the enhanced security of data makes BC perfect for transactions, as users freely benefit from the historical record of data and immediate updating of the current data exchange records, and additionally, the data is not corrupted [28,29,30,31,32]. BC decentralization ensures zero vulnerability to tampering as information once added to the BC can never be changed [12,22,23,24,25,26,27,28,29,30,31,32,33,34,35]. 

BCs can be classified as public BCs (external users have the ability to read and add entries to the ledger), private BCs (the owner determines the rights to blocks and the ability to add and confirm BC transactions) or corporate (contains a pool of selected nodes that allow data to be added to the chain and their reading can be open or private) BCs depending on certain rules, such as authentication and access control techniques [36]. Taking into account the above network classification and taking into account the nature and type of key data for production systems, it is reasonable to use a corporate network, i.e., containing public and private features, whose operation is carefully planned. 

In the area of production management, BCT is not popular. It found interest only among companies operating in the supply chain, mainly due to the possibility of integrating technologies with supply processes [37]. In the production part of the supply chain, there were few implementations of the technology, mainly in the area of creating a production knowledge base and its exchange in order to increase productivity, and with the supply chain, including transactions on self-executing smart contracts, which in turn act as a public database for product tracking [38]. Enterprises have already made significant investments, especially in BC applications supporting payment tracking and product processing and traceability using code tags [39,40]. The service-oriented production information exchange platforms used so far are made available mainly in large-scale data transmission areas, which are associated with the participation of additional entities that manage data through server logistics. 

Data centralization is a convenient solution, but it requires considerable financial and hardware expenditures and energy, and additionally increases the distance between the client and the service. In order to eliminate these problems, central databases began to be replaced with decentralized architecture, i.e., a cloud in cyberspace with controlled access and security of shared data. A solution devoid of data centralization works especially with high process dynamics, delay variability and detailed throughput, i.e., in a dynamic production environment. The use of BCT enables users to record contracts in self-executing smart contracts without additional intermediaries and provides a guarantee of trust in publicly announcing machine resources and the services they provide. The potential of using BCT in production is shaped by factors such as [41,42,43]:

- The ability to join the network of all companies, regardless of their range and size,
- Strong cooperation between contractors (data decentralization, equality in data management, mutual trust between network participants),
- Functioning of BC in IoT (data autonomy),
- Possibility to create new business models,
- Exclusion of unnecessary intermediaries,
- Savings in the search for contractors,
- Easier load modification and optimization of production resources,
- Concluding smart contracts (improvement of efficiency in managing data resources, connection to a code generator).

Limitations that may appear in the implementation of the proposed solution mainly concern [44,45]:

- The number of correctly defined users of the block network (the presence of as many participants as possible is necessary, which will create new nodes and a rich database),
- Scalability,
- High energy and computing power,
- High costs of IT systems configuration,
- Lack of legal standardization for BCT users,
- Performing consensus protocols every time to maintain the integrity of the BC.

A comparison of the flow of information about production tasks and machine resources in the traditional and BC systems is presented in [43].

### 1.2. Goals and Approaches

The main purpose of the article is to adopt an architecture for a data exchange network between manufacturing companies [46,47], as a shared data register with particular emphasis on machine resources. A parallel goal is to develop an algorithm for an efficient division of machine resource occupancy including historical information on resource competences, reliability and information on planned availability between users participating in the exchange of announced and sought-after resources for internal production tasks. 

The authors’ analysis shows that this is the first article in which the implementation of production planning and machine resource management using BCT was developed.

The work consists of four sections. The first discusses the BCT, definitions, general architecture of BCT and its connection with the IoT. The use of BCT in production engineering and positive and negative factors that affect the functioning of BCT in the area of industrial production is presented. Section 2 contains a detailed description of the analyzed example, a proposal for information flow in the chain of connections and the concept of data circulation architecture within shared registers. Section 2.2 presents a research problem and the algorithm of efficient distribution of resources. An example of research simulations and their results are presented in Section 3. Section 4 presents a discussion taking into account the advantages and disadvantages of the architecture of BCT and the algorithm in the numerical example. Section 5 is a summary of the analyzed issue, a presentation of the research conclusions and a proposal for further work.

## 2. Production Planning Method Based on Blockchain Technology

This section presents the motivation for using BCT and the method of applying it to the production planning problem.

### 2.1. Motivation of Using Blockchain Technology in Production Planning 

**Figure 1:**
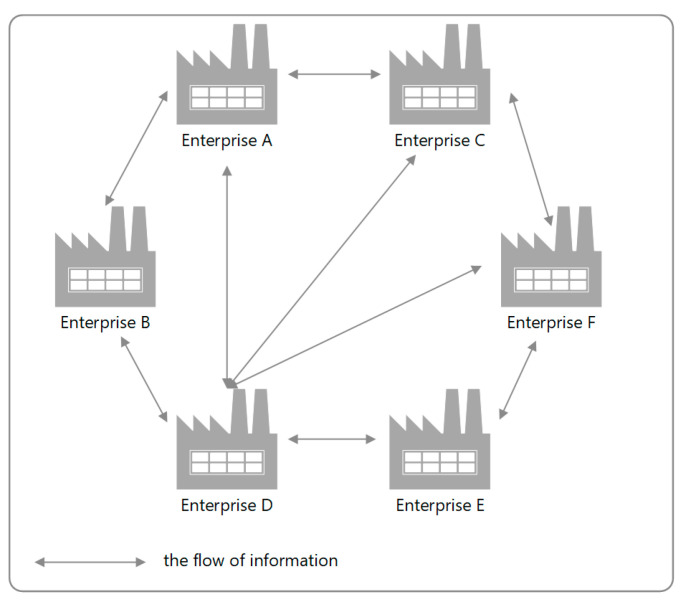
P2P network diagram.

BC is a decentralized computer network that does not have a central database management unit and consists of a chain of blocks [8,48]. The essence of BC is to keep a distributed common and collective register of data records in digital form, which are successively announced in the network equally for each recipient, in accordance with their access. It works on the principle of a global data book, which stores all the records placed in it. These records (transactions) have a time stamp, a cryptographic hash and are combined into a linear structure of blocks, in which each subsequent record refers to the hash of the previous block. An important aspect of BC transactions is their irreversible nature, as there is no real possibility of cyberattacks. The BC architecture enables participants to share a data book through peer-to-peer (P2P) duplication, i.e., a model of communication in the Internet network in which data exchange units (computers) share resources without the need to pass these data through additional servers, and each node of this network is equivalent and has the same permissions. Data is sent and received in a manner equivalent to predetermined priorities (in this solution, each user is a host) [49]. The diagram of the P2P network is shown in Figure 1.

**Figure 2:**
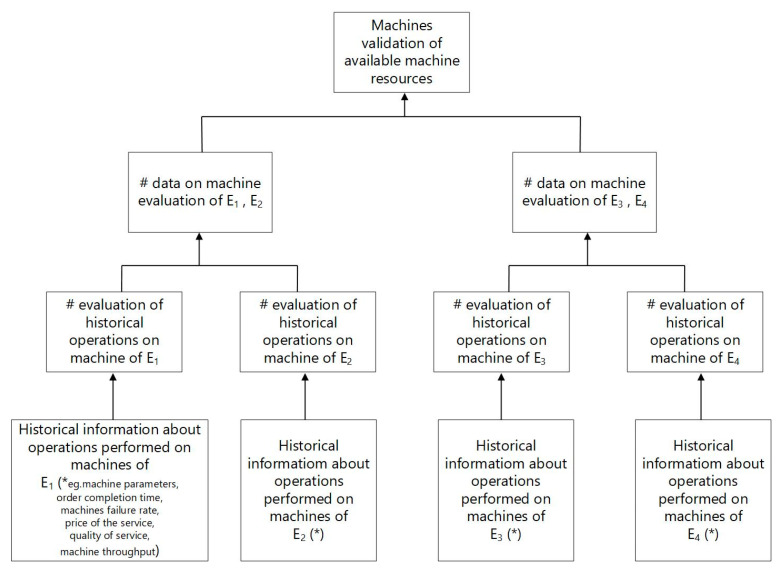
Merkle tree developed on the basis of the analyzed example (“#”—cryptographic hash).

The transactional data in the block body are organized according to a classical data structure in cryptography called a Merkle tree (or Hash tree). In the Merkle tree (Figure 2), the data are grouped every two blocks and create a data structure with two hashes for each group. Each hash pointer corresponds to one block of data. Newly created data structures create the next level of the tree. In the next step, these data are paired again and follow the procedure as before to create the next level of the tree. After such a process, a binary tree is created. The Merkle tree is able to prevent the malicious modification of all transactional data [50]. The issue of cyber defense is provided by BC thanks to its ability to prevent unauthorized activities and thanks to its immutability, auditability and operational resilience.

BC transactions are irreversible, as when BC nodes in the process of verifying transaction changes notice that the data entered does not match previous records in the network, they will refuse to enter them into the chain. The flow of information facilitates enterprise resource planning and enables the integration of internal processes. A fully prepared single block contains information such as: ID—transaction identifier, content sender, content recipient, time stamp, digital signature made by the user’s node, information about added or sought production resources (production parameters, technical data of machines, machine reliability, machine park availability), the criterion for selecting the searched data, a reference to the previous block in the block chain (the hash of this block ensures that the blocks are linked into a chain), the hash of the current block (generated from the data contained in the block, most often using the Merkle tree (Figure 2)), and the list of machines provided by companies E_1_, E_2_, and E_n_ are validated in parallel on the basis of historical qualitative and quantitative data of machine resources. 

**Figure 3:**
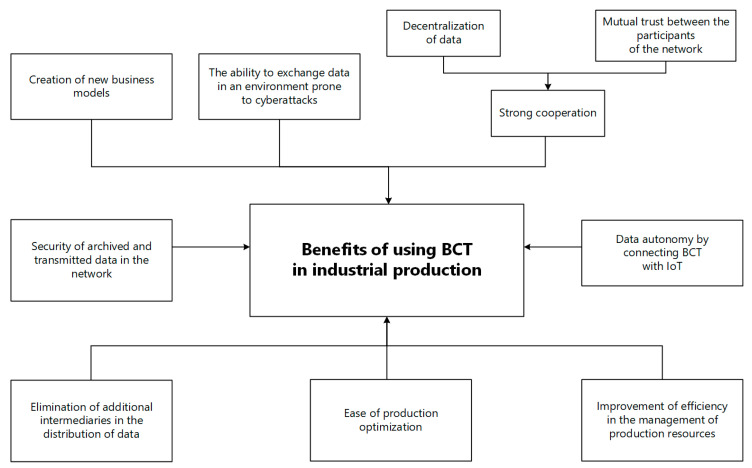
Graphical interpretation of the benefits of BCT implementation.

The most commonly used consensus algorithms in BC are: proof of work (PoW), proof of stake (PoS) and digital signatures based on the public key (which allows authentication of transactions between nodes in the network). Taking the PoW algorithm as an example, spoofing a non-existent record is possible if more than 51% of settlement nodes in the entire network come to an agreement. Such a scenario is basically impossible, because nodes that are constantly joining the BC make counterfeiting impossible, because for a successful attack, the content of each block would have to be forged on an ongoing basis [50,51]. Asymmetric cryptography allows the exchange of information to be encrypted without the need to establish a security key in advance. In BC, it is used for every transaction (i.e., data exchange). The consensus concluded automatically by the participants of the BC network eliminates the need for the presence of a trusted third party when processing transactions. The use of the shared data registered enables quick access to the necessary data in order to optimize the selection of production resources, automate the implementation of production operations, guarantee the reservation of resources (transactions), ensure data security and confidentiality and the possibility of developing the structure of related enterprises and employment under joint production projects. Figure 3 contains a graphical interpretation of the benefits of implementing BCT in managing industrial production.

### 2.2. Production Planning Network 

IoT systems establish the mapping of connections from the real world to the digital world using back-end services and front-end computing devices. Front-end devices are, for example, embedded computer systems equipped with sensors, (temperatures, RFID tags/readers, wearables, flame detectors, cameras, mobile phones, etc.). RFID technology makes it possible to reduce numerous information gaps, mainly in logistics processes, because radio readers provide insight into supply chains in real time [52]. RFID technology can provide automatic traceability, enabling businesses to link objects of the physical world with their virtual counterparts. This traceability method must use a temporal data model to support product traceability and process monitoring [53]. The devices may be in open environments that are beyond the control of the system administrator. The back-end system is a software system that integrates, processes and analyzes the detected information returned by front-end devices, and has the ability to transfer the analyzed results to interested users [54]. For the purposes of the considered simulations, a network of virtual enterprises is created. The ability of data encryption, i.e., the use of cryptographic solutions, is very important for the protection of production data. 

A shared register of machine occupation based on BCT solves most of the existing problems in the area of production resource management. It is hard to trust a potential future competitor and take advantage of its resources; however, BCT guarantees full data protection. It is the users themselves who decide to whom and to what extent they allow insight into the analyzed parameters. 

**Figure 4:**
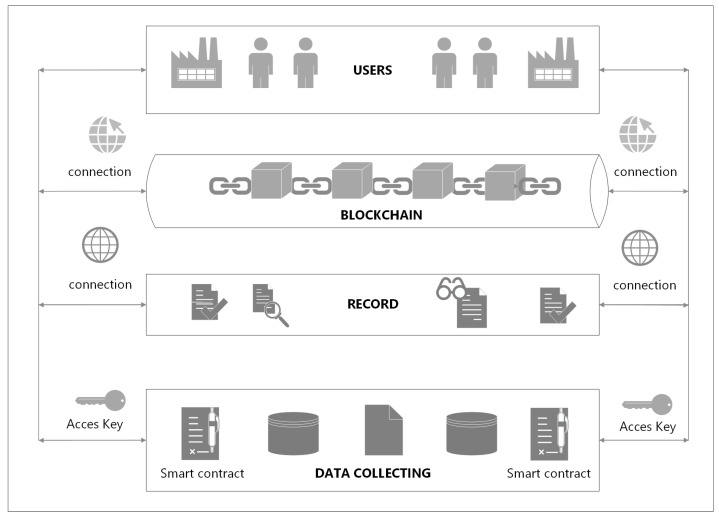
The process of registering data on production resources in the block network.

**Figure 5:**
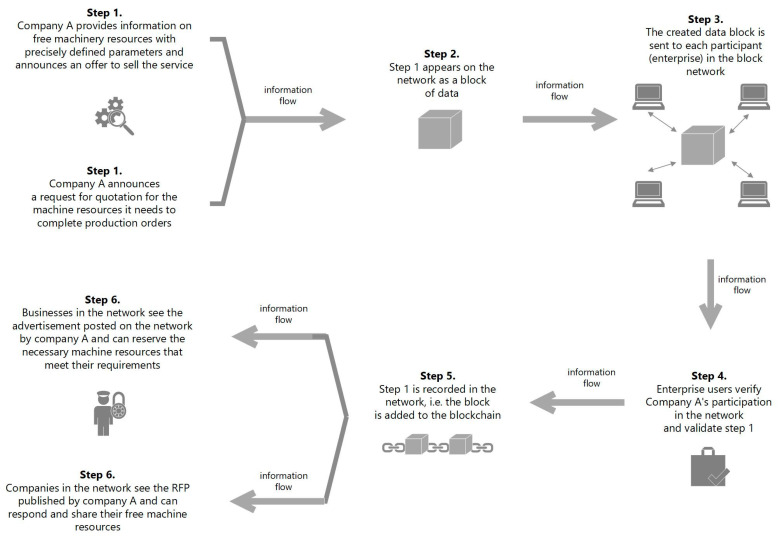
Scheme of BCT operation in the analyzed example, announcement of free resources (the upper line), and searching for free resources (the bottom line).

**Figure 6:**
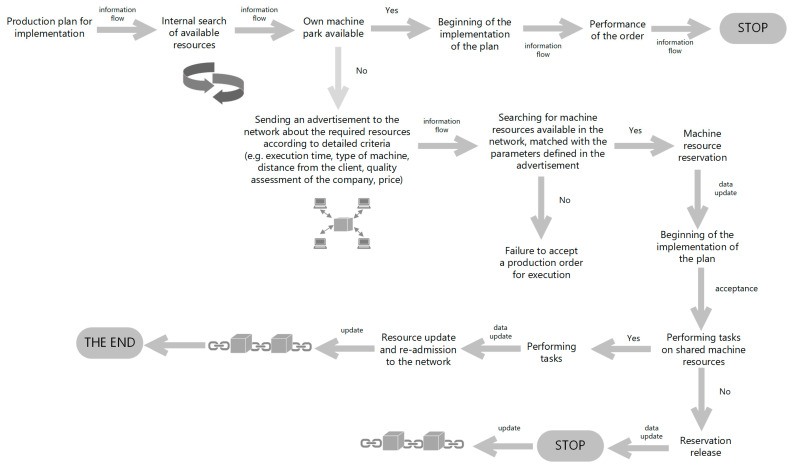
Circulation of information on making free resources available or searching the database of shared registers for their use.

In Figure 4, the process of registering data on production resources in the block network is shown. The information to be recorded is: name of the company, current occupancy of machines, list of machines to be used, type of operations that can be performed on machines, determination of the throughput level, description of operations that were previously performed on machines, price for the service, detailed specification of technological operations to be performed, etc. The collected information is subject to acceptance by network users through a smart contract and is subsequently placed in a collective database from which enterprises can view, reserve and outsource the operations they need, then these companies take the position of a reseller of resources (the bottom line, Figure 5). The same operations take place when the company is looking for free resources to perform certain production activities, then it assumes the role of a customer (the upper line, Figure 5). The flow of information regarding the provision of free resources or searching the shared register database in order to use them is presented in Figure 6.

During the initiation of a request for quotation, the stakeholders are connected by a network of information flow about production machines, resource data and the content of inquiries and responses. For example: when sending a query or announcing free resources, the system will first request the user’s identity to make the information available on the web. The authentication request is sent to all interested parties (see step 3, Figure 5). Only after verifying these data will the system make the found records available (matched according to the criteria set in the query).

**Figure 7:**
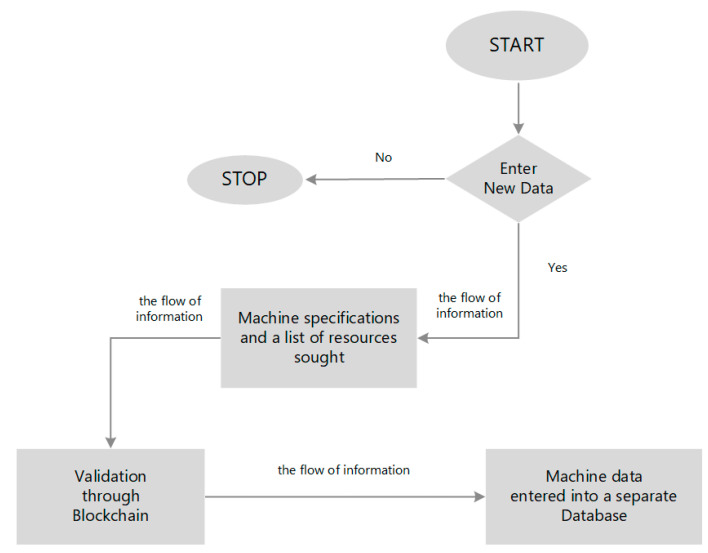
The stages of the process of entering and functioning of data in the network.

A query initiates the process of entering new data (or extracting data already existing in the database) regarding the specification and availability of machines. In the absence of the requested data (machines with a specific specification), the process is considered completed. If the company decides to introduce new information to the network, a block with a full specification of machines is created. Subsequently, these data undergo a validation process (documentation in accordance with predetermined rules). After successful acceptance by other network participants, they are entered into a dedicated database. Each machine contains a set of the same features that appear in the main database and in the available/unavailable status. The stages of the process of entering and validation are shown in Figure 7. The criterion of maximum use of the existing machine resources of enterprises in the BC network, such as makespan, is validated. 

The main idea of the method is to define a set of basic network nodes and basic interactions between them. Since users can add their own segments to the system and expand existing nodes, the information exchange platform becomes an optimal distributed system after some time based mainly on the requirements and needs of users and enables the exchange of information about the current state of machine in real-time. 

The first node, the Genesis node, implemented as a smart contract, contains essential information about the network, acts as an entry point for new users and a guide for creating new nodes. The Genesis node has information about the location of all network nodes.

The next nodes are network users who wants to access the platform in order to cooperate with other platform nodes. A manufacturing company wants to share its machine resources or is looking for machines to carry out its production plan (Figure 5). Decentralized production management is mainly used to properly optimize production. They face the challenge of deciding to outsource the task (they do not have the appropriate machine infrastructure in their resources) or to performing tasks from other companies (for an additional fee).

In order to enter information about the needed resources and search the database, the Genesis node is crucial. The Genesis node provides a link to all the underlying nodes of the BC. When a company is looking for available machine resources, attention is focused on mapping companies with free resources (which previously reported such readiness by adding information to the BC network). In addition to the list of all parameters that are necessary to complete the order, the entered information also determines the access time, the type of delivery and transport of semi-finished products and parts, payment details, process details, etc. It is also worth specifying the area if the transport of products is involved in order to make a real assessment of the financial profits. When the company receives a list of all the registered suppliers in the chain that meet the listed conditions, it starts sending its order to all those who have expressed their willingness to provide machines. Enterprises return their bids back to the BC, and the client company can choose the most advantageous offer for its production.

When looking for an effective algorithm for assigning tasks to machine resources, the following conditions are important: experience (quality of operations performed on the machine), reliability (machine with a second life cycle), as well as the cost of outsourcing and efficiency for the planned working time horizon. Reliable historical data on failure-free times are essential to accurately machine uptime predictions. In addition, the machine with the second life cycle is characterized by the constant failure-free operation, which allows the managers to build stable and robust schedules [55]. The production planning algorithm based on the BCT is presented in the next sub-section.

### 2.3. Production Planning Algorithm

**Figure 8:**
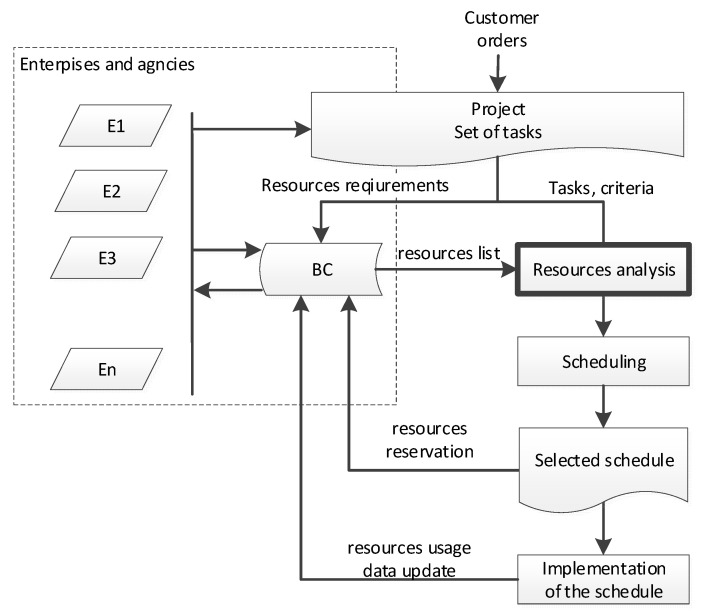
Resource specification data flow in the scheduling process.

Task planning algorithm along with multi-criteria optimization mechanisms as well as mechanisms enabling supplying these algorithms with relevant, up-to-date data are necessary. The production scheduling process with the use of BC technology to manage distributed machine availability is summarized in Figure 8.

Accepting an order for scheduling requires designating a set of available machines that can be used for its execution. For this purpose, an inquiry is made to the BC registers of all partners participating in the BC network. Machines with the required specifications are searched. The main assumptions are:

- An operation requires more than one resource to be reserved, e.g., groups of employees, machines,
- The operation requires the reservation of employees with different competencies (workers with different experience and competency) [43],
- The operation requires the reservation of a machine with specification (machine type + reliability characteristics of machines + tools + distance between companies, cost of renting a machine or cost of performing an operation),
- The operation needs to be executed in order to achieve the best performance indicators.

Each shared machine should have its Genesis node initialized. In terms of information stored in the GB block, required for planning activities, the specifications of the machine (in qualitative and quantitative terms) are recorded along with their valuation and the working time calendar. The individual periods (Om_i_) are described by the following:

Om_i_ = (tb_i_, te_i_, E_m_, λ_m_, C_m,_ P_m_, u_i_), (1)

where:

i—the index of the work period,

tb_i_, te_i_—start and end time of the work period, respectively,

E_m_—experience (quality) in using machine m, 

λ_m_—reliability of machine m, 

C_m_—cost (distance) of the machine using,

P_m_—performance of the machine using,

u_i_—generalized value of the machine execution evaluation, u_i,k_ ∈ <0;1>.

The generated machine list is used for scheduling. Having the optimal schedule, we also have the start and end times of the work period for machine m. Since the BC contains all the historical activities of machine m, it is possible to use it to determine the current value of the machine within each of: experience (quality), reliability (the second life cycle of a machine), as well as with outsourcing cost and performance for the planned horizon.

The registration of the above data enables a later review of the machine and operator work history and determination of their current value within individual features. The machine operator experience valuation method is:

(2) $$\vartheta_{z,m}^{\mathrm{tn}}=\sum_{T_{1;\;\mathrm{im}}}^{T_{2}}\left(\;\mu \left({\mathrm{te}}_{i,m}\right)\;\left({\alpha \;v}_{i,m}+\beta \frac{\left(({\mathrm{te}}_{i,m}+1)-{\mathrm{tb}}_{i,m}\right)}{\sum_{T_{1;\;i}}^{T_{2}}\left(({\mathrm{te}}_{i}+1)-{\mathrm{tb}}_{i}\right)}\right)\right),$$

where:

i, n, x—time, 

$\vartheta_{z,m}^{\mathrm{tn}}$—assessment of machine operator m in terms of quality of executed products, on time production, and experience in time t_n_,

v_i,m_—evaluation of the i-th period of work with competence m,

$t_{n}$—the moment of determining the grade,

tb_i,m_, te_i,m_—start and end time of the work period with machine m, respectively,

T_1_, T_2_—beginning and end of the considered period of historical data analysis,

$\mu \left({\mathrm{te}}_{i,m}\right)$—coefficient of the work period value at time ${\mathrm{te}}_{i,m}$, 

α—importance coefficient of the work quality, α ∈ <0;1>,

β—importance coefficient of duration time of work (experience), β ∈ <0;1>.

The machine availability evaluation method is:

(3) $$u_{z,m}^{\mathrm{tn}}=\left(1-e^{-\lambda_{i,m}{\mathrm{te}}_{i,m}}\right)\;\left(\gamma \frac{d_{i,m}\left({\mathrm{te}}_{i,m}\right)}{{\mathrm{Max}\;d}_{i,m^{*}}}+\varphi \frac{1}{\vartheta_{z,m}^{\mathrm{tn}}}\right),$$

where: 

$u_{z,m}^{\mathrm{tn}}$—assessment of machine m in terms of operator experience, reliability and cost in time t_n_,

$\lambda_{i,m}$—parameter of machine reliability in the i-th period of work estimated based on historical periods,

$d_{i,m}$—the distance (or cost) of outsourcing the machine,

$d_{i,m^{*}}$—the machine for which distance (or cost) is maximum,

Γ—importance coefficient of the distance, γ ∈ <0;1>,

Φ—importance coefficient of duration time of work (experience), φ ∈ <0;1>, moreover γ+ φ=1.

According to the above Formula (2), the evaluation of the value of the operator experience at time t_n_ is based on historical data within a fixed range 〈$T_{1},{\;T}_{2}$〉, considering the time that has elapsed since the activities performed within competence k, using µ(t_x_). This allows for the differentiation of partial assessments and a preference for the quality that have been acquired recently, which is particularly important if a given enterprise improves the quality of his work over time. An example of calculating the value of an experience of a single enterprise is presented in Table 1.

In this example, the registered historical period was assumed to be 12 time units, the values of the µ(t_x_) coefficient in the range (0.3–1), decreasing linearly with time (older periods have a lower coefficient). The coefficient of the work period value µ(t_x_) at end time t_x_ = te_ik_ of competence k of the machine operator is calculated. Two activities related to experience k = E_1_ were registered and assessed in the employee’s schedule: Oe_1_ = (2,4, C_1_,0.7) and Oe_2_ = (8,9, C_1_,0.9). The values of 0.7 and 0.9 were reached during the evaluation after each activity on machine m (including quality and on time production). The final assessment of the competence of machine operator E_1_ is 1.609 for the assumed values of α = 1 and β = 1.

According to the Formula (3), the evaluation of the value of machine availability at time t_n_ is also based on historical data on failure-free times within a fixed range 〈$T_{1},{\;T}_{2}$〉. The range 〈$T_{1},{\;T}_{2}$〉 is divided into 12 periods, for which failure-free times were extracted using BC. The parameter of exponential distribution is predicted for the next scheduling period 〈$T_{3}=13,{\;T}_{4}=24$〉. λ is estimated for each historical period using (4) and the predicted value is substituted into Formula (3). The predicted $\lambda_{i+1,m}^{}$ of machine M_1_ is 0.007 for the assumed values of γ = 0.5 and φ = 0.5.

(4) $$\lambda_{i,m}^{}=\frac{n_{i}}{\sum_{k=1}^{n_{i}}x_{i,k}},$$

where: x_i,k_—failure-free time of machine m registered in the ith period, n_i_—number of failure-free times of machine m registered in the ith period.The reliability of the machine is read for the planned completion time of the operation. The second part of the Formula (4) is the distance (or cost) of outsourcing the machine/operation request, divided by the maximum distance from all enterprises that respond to the request. The last component of the equation is the inverse value of the experience of the machine operator, since Equation (4) tends to a minimum. The final assessment of machine availability M_1_ is 0.071 for the assumed values of γ = 0.5 and φ = 0.5. The example of calculating the value of machine reliability using Equation (4) is presented in Table 2.

The value of a machine in terms of operator experience and availability is obtained from periods representing work history (BC). A machine must be available to engage in a task. If there are more resources available than the requirements of the planned task, a greater number of variants may arise at the scheduling stage. The created schedules require evaluation and selection of a machine for implementation, taking into account performance indicators (makespan, total tardiness).

The information on failure-free times of the machine as well as the life cycle stage of the machine is also obtained from periods representing work history (BC). The machine reliability is described by the Weibull distribution. In the case of the second life cycle, the Weibull distribution (with the scale parameter equal to 1) takes the exponential form (the first component of Equation (3)). The machine availability is reduced when the reliability of the machine is lower (Table 1). 

The selection of the schedule variant for implementation requires registration in the BC network due to the necessity to reserve terms for selected resources. Then, upon completion of the scheduled tasks, the BC resources are updated with a new period (1) with a current assessment of the enterprise experience. The process of machine selection is repeated for another task.

After reaching a mutual agreement on the implementation of the resource occupation, the details are written on the smart contract of the Agreement node. In this network location, the manufacturing company and the company providing their machine resources sign a contract (virtual, for this purpose they use their own digital signatures). Machine resources used during this project are blocked on the smart contract. After completing the task, the parties report the result to the blocks, if they agree as to the correctness of implementation and fulfillment of all production criteria, payment for the implementation is made on the terms previously defined. In the event that all parties to the contract cannot agree, the funds are blocked in the contract. The performance evaluation node can then take this into account when determining trustworthy users. It is worth noting that each order package has its own user node and has the ability to interact with other nodes on the platform. Bundle nodes are programmed to meet criteria set by a company wanting to take advantage of another company’s free machine resources. 

If a company wants to perform operations in the cheapest possible way, the network is searched for the cheapest offers from service providers. In order to ensure the best quality of the product, the network will be searched to find the conditions of the best quality machines offered by service providers. What’s important: companies can generate their own smart contracts that will specify the nodes of mutual agreements. The new smart contract may be responsible for providing information only about machines with specific quality certificates.

## 3. Numerical Example

The example of selecting resources for a task using the mechanisms described in the article is presented below. A resource is sought for a task (Operation) requiring C1 competence, for the period of 15–17 time units. The decision time is set at 12. 

**Figure 9:**
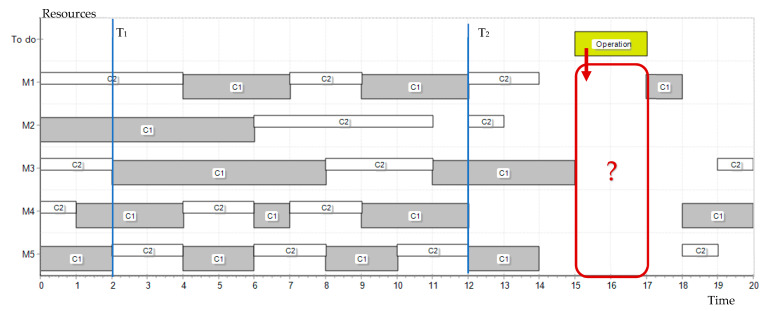
The problem of selecting a resource for an operation—range of resource work periods evaluation.

Based on the reported resource requirements from the BC, information on five resources (M1–M5) was obtained. Figure 9 shows the history of their work (0–12) and the current assignment of tasks and reservations (12–20). It was also assumed that the last 10 time units will be taken into account for the history analysis. The resources work alternately according to two competences, C1 and C2, but the assessment is carried out only in the area of the required competence.

During the conducted analyses and verification of the adopted assumptions, proprietary software was used: based on the project [56]—in the field of communication and support of block chains in the consortium network, and the kbrs program [57]—for scheduling and preparing information for block coding.

Table 3 presents data based on information from the BC: resource work schedule (presented above) according to relevant competencies and task evaluation values (v_i,m_). Individual periods of work were assigned a value (coefficient of the work period value at time) in the range of 0.3–1. The last column presents the summary assessment for only the periods of work in the competency sought (C1).

Table 4 presents the partial and the final results of resource selection. The calculations were based on the weights for the coefficients of the work quality, experience, distance (cost of access to the resource) and general assessment of competence, respectively: α = 0.5, β = 0.5, γ = 0.5, and φ = 0.5. In each decision situation, the values of these parameters may be different. The values of the $d_{i,m}$ parameter were adopted arbitrarily, and the $\lambda_{i}$ column presents the final calculation results for the given input data.

According to the formulas presented in this work, the minimum value of the evaluation is sought. Therefore, the resource reservation, resulting in the creation of the next block in the resource BC, will be reported to resource M1. After performing the tasks planned in the schedule, the resource registers are updated with the current assessment of their work. 

## 4. Discussion

The presented numerical example relates to the stage of analysis and selection of resources for the implementation of planned tasks. This is a key step, listed in the data flow diagram (Figure 8) as “Resource analysis”. 

**Figure 10:**
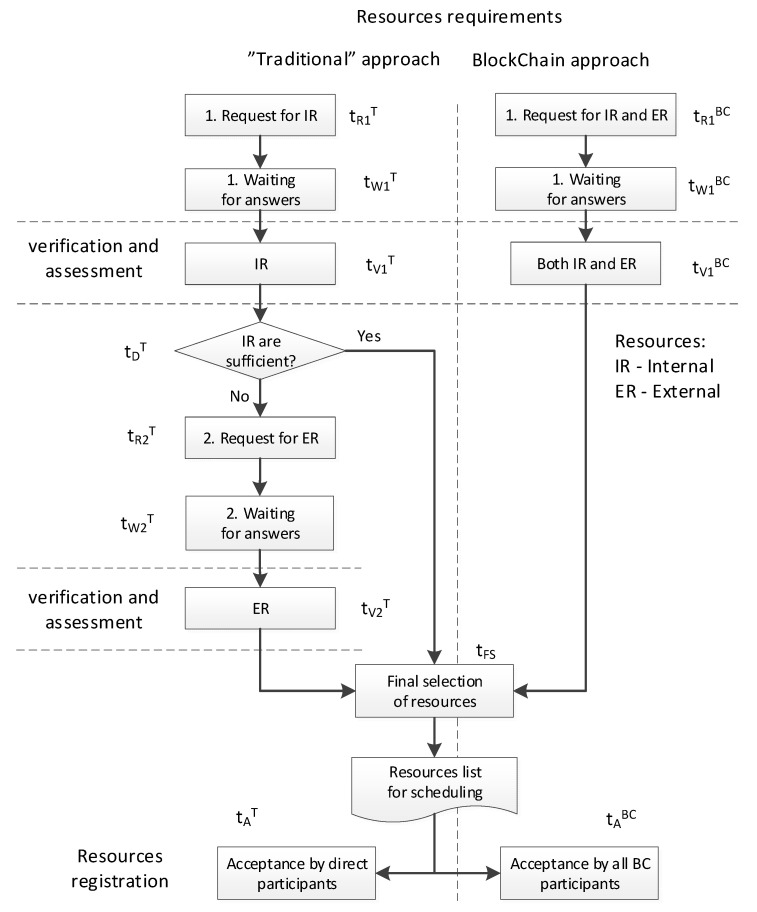
Traditional and BC-based approaches for resources selection.

In relation to the traditional approach, when selecting resources, the ability to immediately and simultaneously analyze all available resources, both internal (in own enterprise) and external (enterprises in the consortium), allows for significantly reducing the time and choosing the best available resources. Figure 10 presents a comparison of the traditional approach with the proposed one based on BC technology. Traditionally, when searching for resources, the organization’s own resources are taken into account in the first place, which is also due to the access to the necessary data—enterprises most often do not exchange current data on the load on their resources. If their own resources are sufficient, tasks are assigned to them. If they are not, or the results of the evaluation of the selected resources are not satisfactory, resources from outside the organization are sought in the next step. Activities related to sending queries and waiting for a response (queries must be processed by partners) and the additional stage of verification and evaluation of external resources increase the number of sequential activities and significantly extend the duration of the decision-making process. The BC approach simplifies these activities as much as possible. Compared to the path in the traditional approach, where the selection of resources ends with internal resources, the proposed BC approach should create an equal or better quality solution—because the selection is made on a wider set of resources. The decision-making stage at which the use of their own resources is decided is also omitted, and which may also have a significant impact on the total duration of resource selection.

Leaving aside less important factors, the time of resources reservation (T) for the needs of future tasks, in the scope considered in this paper, can be described by the general formula:

(5) $$T=t_{R1}^{}+{\;t}_{W1}^{}+{\;t}_{V1}^{}+{\;t}_{D}^{}+{\;t}_{R2}^{}+{\;t}_{W2}^{}+{\;t}_{V2}^{}+{\;t}_{\mathrm{FS}}^{}+{\;t}_{A}^{}{}_{i}$$

where:

t_R1_—time of processing and sending a query regarding directly available resources,

t_W1_, t_W2_—response waiting times, 

t_V1_—time of verification of received data and evaluation of directly available resources, 

t_D_—time to make a decision to use own resources/use outsourcing,

t_R2_—time of processing and sending a request for external resources, 

t_V2_—time of verification of received data and evaluation of external resources, 

t_FS_—final solution selection time, 

t_A_—time of acceptance and confirmation of resource reservation.

The above symbols are written in Figure 10 with superscripts that indicate a given approach (T—traditional, BC—blockchain). Depending on the approach, the individual components of the formula for calculating the duration of resources reservations can be calculated in different ways. The Formulas (6)–(8) presented below concern the calculation of time for the traditional approach in two versions, without and with outsourcing (T^IR^, and T^IE^, respectively) and the BC approach (T^BC^).

(6) $$T^{\mathrm{IR}}=t_{R1}^{T}+{\;t}_{W1}^{T}+{\;t}_{V1}^{T}+{\;t}_{D1}^{T}+{\;t}_{\mathrm{FS}}+\max_{i}\left(t_{A,i}^{T}\right)$$

(7) $$T^{\mathrm{IE}}=t_{R1}^{T}+{\;t}_{W1}^{T}+{\;t}_{V1}^{T}+{\;t}_{D1}^{T}+\frac{\;\sum_{i}t_{R2,i}^{T}}{n}+\;\min\left(t_{L},\max_{i}\left(t_{W2,i}^{T}\right)\right)+{\;t}_{V2}^{T}+{\;t}_{\mathrm{FS}}+\max_{i}\left(t_{A,i}^{T}\right)$$

(8) $$T^{\mathrm{BC}}=t_{R1}^{\mathrm{BC}}+{\;t}_{W1}^{\mathrm{BC}}+{\;t}_{V1}^{\mathrm{BC}}+{\;t}_{\mathrm{FS}}+\max_{i}\left(t_{A,i}^{\mathrm{BC}}\right)$$

where:

i—number of external contractors,

n—number of employees handling resource planning,

tL—response waiting timeout.

In the case of T^IE^ (7), the handling of external requests t_R2_^T^ will be sequential, and its duration can, with some approximation, be taken as the quotient of the sum of contact times with external partners and the number of operators working in parallel. The waiting time for all responses sent to external partners is calculated as the maximum value of waiting times, limited by the fixed maximum time—t_L_, if the counterparty does not want to reply.

Table 5 presents exemplary values of times that were estimated for individual components and a reference to the quality of the solution (sq). Values that usually last less than a minute are rounded up to 1. Assuming a similar level of digitization of order handling processes in the enterprise, it can be assumed that t_FS_ is constant and independent of the applied approach, and t_R1_^T^ = t_R1_^BC^ = 5, t_W1_^T^ = t_W1_^BC^ = 1, t_V1_^T^ = t_V1_^BC^ = 5, for which in real conditions there will certainly be slight differences in plus or minus. Time t_D_ occurs only in the traditional approach, but its value is small, therefore t_D_ = 1 was assumed.

The total T^IE^ result is greatly influenced by the additional calculation cycle t_R2_^T^ + t_W2_^T^ + t_V2_^T^, which in the traditional approach must be run in the absence of availability of an organization’s own resources. It takes a relatively long time, as it requires direct contact with external partners (by phone, e-mail) and requires them to check the calendar of resources with the required competences each time. For various reasons, answers are not always provided immediately. For the same reason, the resource acceptance time t_A_ is also long in this case. The acceptance of only their own resources in T^IR^ and by BC can be taken as immediate. The example does not show the difference in the processing time of T^IR^ and T^BC^—in fact, they can also be comparable, assuming full digitization of the resource reservation process. If, for some reason, BC contracts in the consortium network require “manual” approval, the t_A_^BC^ time may be slightly longer. However, due to access to a wider set of resources, the solutions offered by BC may be of equal or higher quality (sq).

**Figure 11:**
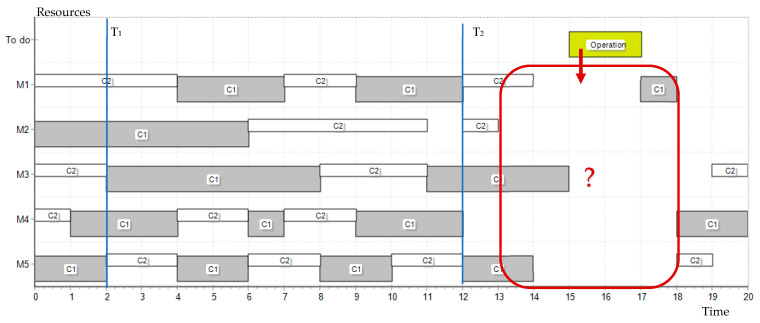
Exemplary extension of the problem (Figure 9), which requires additional consideration of time/cost parameters consideration.

The developed numerical example does not exhaust the issues of resource selection for the scheduling process. It focuses on the basic parameters related to the assessment of the broadly understood history of their work and location, as key in the demonstration of BC technology. In real-life applications, the time interval within which an operation can be planned is often wider than the duration of the operation itself. Figure 11 shows a modification of the resource query in which the possible range for planning operations has been extended.

Depending on the adopted scheduling strategy, recommendations for arranging operations in a given range may be different and prefer the operation as early as possible (A) in the indicated range—with the forward strategy, as late as possible (B)—with the backward strategy, or it may not matter (C)—when it has no impact on other operations related to the planned one. In cases A and B, the assessment of resources should take into account additional parameters related to the start time of operation (backward), completion time (forward), as well as the calculation of the duration/resource operation cost, if they differ for individual resources, etc. However, this does not fundamentally affect the manner in which resources are serviced, booked and evaluated within a group of companies organized in a consortium. Nevertheless, the extension of the model with such elements is the subject of further research.

## 5. Summary and Conclusions

BC is considered to be a revolutionary digital distributed system based on cryptography, i.e., a method of storing and transmitting information on the Internet on the basis of detailed access of participants. In a distributed system, the owner, equipped with a private key, has full control over transactions. Transactions are defined with unique codes that prevent the same unit from being sent twice to several recipients. Importantly, BC is the answer to the need for secure storage and effective data analysis, thanks to the combination of data archiving, cybersecurity, authentication, access only for authorized users and secure and distortion-free data handling. Modern production is more and more often identified with the concept of intelligent production, it is fully integrated and its assumptions are based on close cooperation between entities. This gives users the ability to react immediately to changing requirements in the factory, supply networks and consumers in real time [58,59]. The technology allows for the complete elimination of intermediaries, i.e., entities (or institutions) that have full access to data in standard systems and perform paid operations for a decentralized register. 

This paper proposes a BC-based shared production resource registry project. The technology allows for transparent data exchange and ensures security in cyberspace among the cooperating production networks of many enterprises. The security of transactions is ensured mainly by unique identification marks, transaction authentication by concluding smart contracts and their authenticity. Each network user has a virtual identity corresponding to the physical state of the institution (department, company or consortium of enterprises) they represent.

The presented approach differs significantly from existing commercial Internet solutions. When formulating their requirements and making them available on the network, manufacturers expect credible answers and reliably machines to fulfill orders with the highest quality and data security at the lowest cost, maximum efficiency and the shortest lead time. All these aspects illustrate the importance of an innovative and flexible approach to planning the use of production resources. 

In traditional systems, cooperation usually takes place within several cooperating companies. The demanding consumer market and its dynamism forces producers to change their approach to production management and the implementation of innovative solutions in the field of process planning and scheduling. Most of the emerging problems related to the traditional management model seem to be solved by BCT. 

The attempts to integrate the virtual environment and the real one presented by the authors may soon lay the foundations for the factories of the future, in which it will be possible to manage fully automated and robotic production using completely remote control. Therefore, every effort should be made to create an extremely secure space for process control and data exchange, resistant to cyberattacks, with the highest throughput and the shortest uptime. The proposed architecture supports production and increases its efficiency, and does not delay or slow down processes. According to the authors’ knowledge, this is the first paper to integrate production planning and machine resources management using BCT. The widespread computerization of industrial services and the current era of Industry 4.0 motivate manufacturing companies to follow market requirements and respond to new challenges. In the production sector, there are many technological factors that enable working with a huge amount of business data (automation, robotization, IoT) and bring new challenges, mainly in the field of cyber security [60,61,62,63].

The direction of further research includes attempts to implement BCT in the processes of selling or renting machine resources and other attempts to allocate resources equally and scheduling tasks with constraints. Future proposals include the possibility of fully automated order fulfillment, numerous options for simulation and sequential optimization of all production processes. The authors plan to develop multi-criteria optimization methods to build consensus on the problems of production planning. The authors plan to develop online scheduling algorithms to achieve consensus among consortium members on production planning. 

## Figures and Tables

**Table 1:** An example of calculating the value of a single machine availability using Equations (2) and (3) for γ=0.5 and φ=0.5.

**Historical Period t_x_**	**1**	**2**	**3**	**4**	**5**	**6**	**7**	**8**	**9**	**10**	**11**	**12**	**Sum**
µ(t_x_)	0.300	0.364	0.427	0.491	0.555	0.618	0.682	0.745	0.809	0.873	0.936	1.000	
experience		E_1_	E_1_	E_1_				E_1_	E_1_				
(te_i_ + 1) − tb_i_				3					2				5
v_i,k_				0.7					0.8				
Partial eval.				0.638					0.971				1.609
**Planning Period t_x_**	**13**	**14**	**15**	**16**	**17**	**18**	**19**	**20**	**21**	**22**	**23**	**24**	
$1-e^{-\lambda_{i,m}{\mathrm{te}}_{i,m}}$ machine $\frac{d_{i,m}\left({\mathrm{te}}_{i,m}\right)}{{\mathrm{Max}\;d}_{i,m^{*}}}$ 1/Sum of partial eval Final eval.	0.087	0.093	0.100	0.106	0.112	0.118	0.125	0.131	0.137	0.143	0.149	0.155	
				M_1_	M_1_	M_1_	M_1_						
							0.525						
							0.621						
							0.071						

**Table 2:** An example of calculating the reliability of the machine using Equation (4).

Historical Period	No.	1	2	3	4	5	6	7	8	9	Sum	λ_i_
1	5	150	180	200	230	240					1000	0.005
2	6	100	140	150	190	210	200				990	0.006
3	6	50	100	150	150	280	250				980	0.006
4	4	100	250	250	300						900	0.004
						…						
12	8	80	100	120	125	135	140	140	150		990	0.008
Planned period											predicted	0.007

**Table 3:** Calculations of competency values for M1-M5 resources.

	Period (i)	3	4	5	6	7	8	9	10	11	12	Sum
	µ(te_i,k_)	0.5	0.556	0.611	0.667	0.722	0.778	0.833	0.889	0.944	1	Comp = 1
M1	Comp	2	2	1	1	1	2	2	1	1	1	
	v_i,m_	0.5	0.8	0.8	0.5	0.7	0.5	0.6	0.9	0.9	0.8	
	u_i,m_	0.150	0.250	0.275	0.200	0.289	0.233	0.292	0.444	0.472	0.450	2.131
M2	Comp	1	1	1	1	2	2	2	2	2	-	
	v_i,m_	0.6	0.7	0.6	0.6	0.7	0.5	0.6	0.8	0.8	-	
	u_i,m_	0.175	0.222	0.214	0.233	0.289	0.233	0.292	0.400	0.425	-	0.844
M3	Comp	1	1	1	1	1	1	2	2	2	1	
	v_i,m_	0.5	0.5	0.5	0.6	0.6	0.6	0.7	0.7	0.8	0.7	
	u_i,m_	0.150	0.167	0.183	0.233	0.253	0.272	0.333	0.356	0.425	0.400	1.658
M4	Comp	1	1	2	2	1	2	2	1	1	1	
	v_i,m_	0.7	0.7	0.6	0.5	0.6	0.5	0.5	0.6	0.7	0.7	
	u_i,m_	0.200	0.222	0.214	0.200	0.253	0.233	0.250	0.311	0.378	0.400	1.764
M5	Comp	2	2	1	1	2	2	1	1	2	2	
	v_i,m_	0.7	0.8	0.7	0.7	0.7	0.7	0.8	0.8	0.7	0.8	
	u_i,m_	0.200	0.250	0.244	0.267	0.289	0.311	0.375	0.400	0.378	0.450	1.286

**Table 4:** Resources values calculation.

	$d_{i,m}$	$\lambda_{i}$	$\vartheta_{z,m}^{tn}$	$\frac{d_{i,m}\left(te_{i,m}\right)}{Max\;d_{i,m^{*}}}$	$1/\vartheta_{z,m}^{tn}$	$e^{-\lambda te_{i,m}}$	$1-e^{-\lambda te_{i,m}}$	$u_{z,m}^{tn}$
M1		0.006702	2.131		0.469361	0.99332	0.00668	0.001568
M2		0.007143	0.844		1.184211	0.992883	0.007117	0.004214
M3	1	0.007535	1.658	1	0.603015	0.992493	0.007507	0.006017
M4	0.5	0.006944	1.764	0.5	0.566929	0.99308	0.00692	0.003692
M5	0.6	0.007937	1.286	0.6	0.777538	0.992095	0.007905	0.005445

**Table 5:** List of estimated components of resource reservation time (min) and solution quality.

Approach	t_R1_	t_W1_	t_V1_	t_D_	t_R2_	t_W2_	t_V2_	t_FS_	t_A_	Sum(T)	sq
IR	5	1	5	1	-	-	-	2	1	15	sq^IR^
IE	5	1	5	1	5	20	5	2	15	59	sq^IE^ ≥ sq^IR^
BC	5	1	5	-	-	-	-	2	1	15	sq^BC^ ≥sq^IR^

## Data Availability

Not applicable.
